# Supplementary figures and images for: UltraPrep is a scalable, cost-effective, bead-based method for purifying cell-free DNA
Source: PLoS One. 2020 Jun 1;15(6):e0231854. doi: 10.1371/journal.pone.0231854 (PMC7263608; doi:10.1371/journal.pone.0231854)

## Slide 1
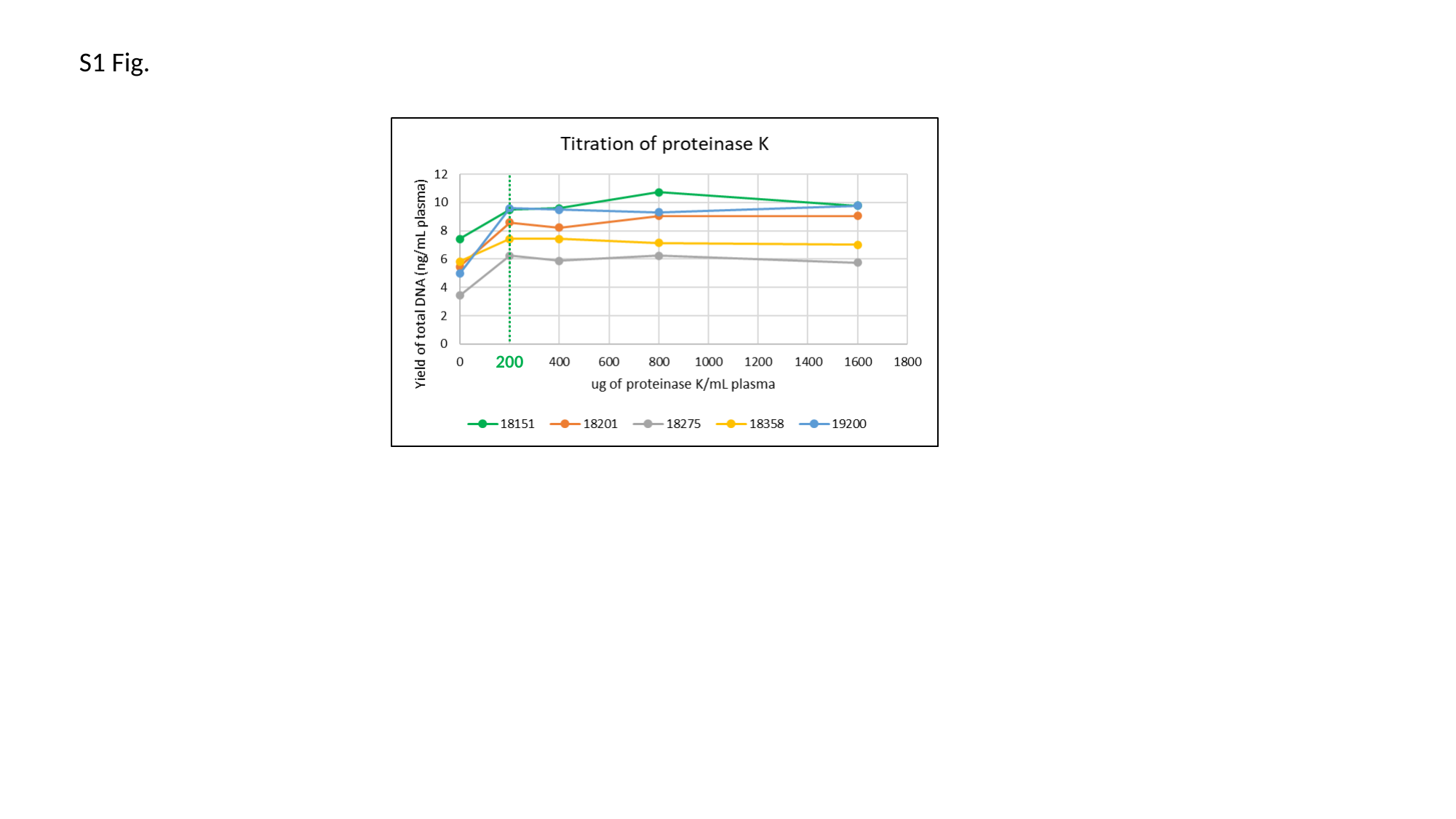

S1 Fig.
200

Supplement: S1 Fig — Identical 10 mL aliquots of several different donor samples were processed using concentrations of proteinase K shown. The quantity of 200 ug/mL plasma (10 ul of a standard 20 mg/mL solution of enzyme added per mL of plasma) that was chosen for the protocol is highlighted in green. This amount of enzyme corresponds to four cents per mL of processed plasma, which is less than 10% of the overall cost. While there were significant yields of DNA in the no-added-enzyme control samples, these samples were “sticky” and extremely difficult to process. (PPTX) [file pone.0231854.s001.pptx]

## Slide 1
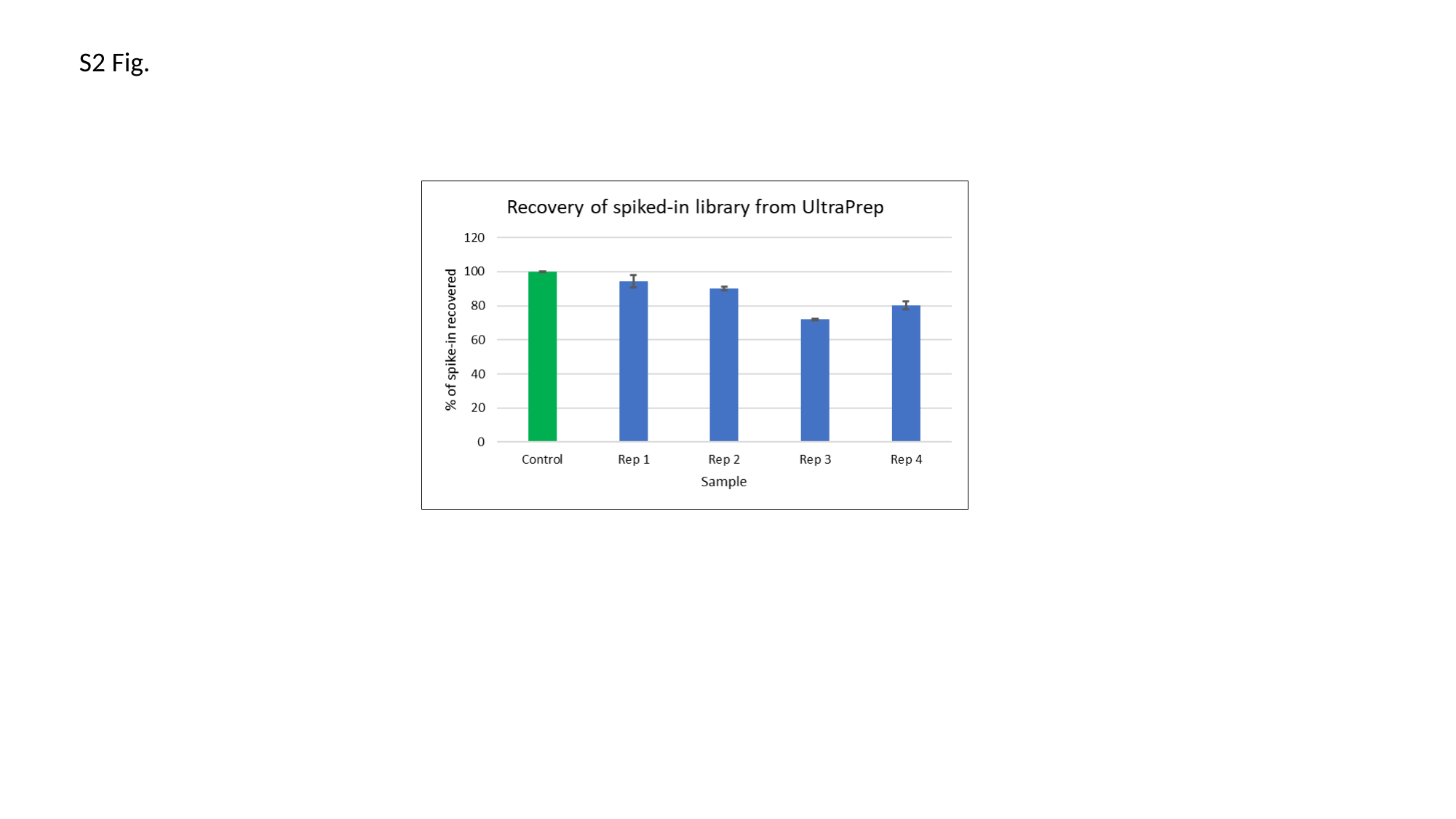

S2 Fig.

Supplement: S2 Fig — The spike-in DNA was a completed cfDNA library with Illumina adapter sequences. This DNA can be specifically detected in a qPCR reaction by using an Illumina P5-specific primer coupled with a human Alu primer (see Methods). One ng per mL of plasma was added. The amount of library in the preprocessed control and four replicates was determined by qPCR using the Illumina + Alu primer pair. The average recovery from the four samples was 84%. (PPTX) [file pone.0231854.s002.pptx]

## Slide 1
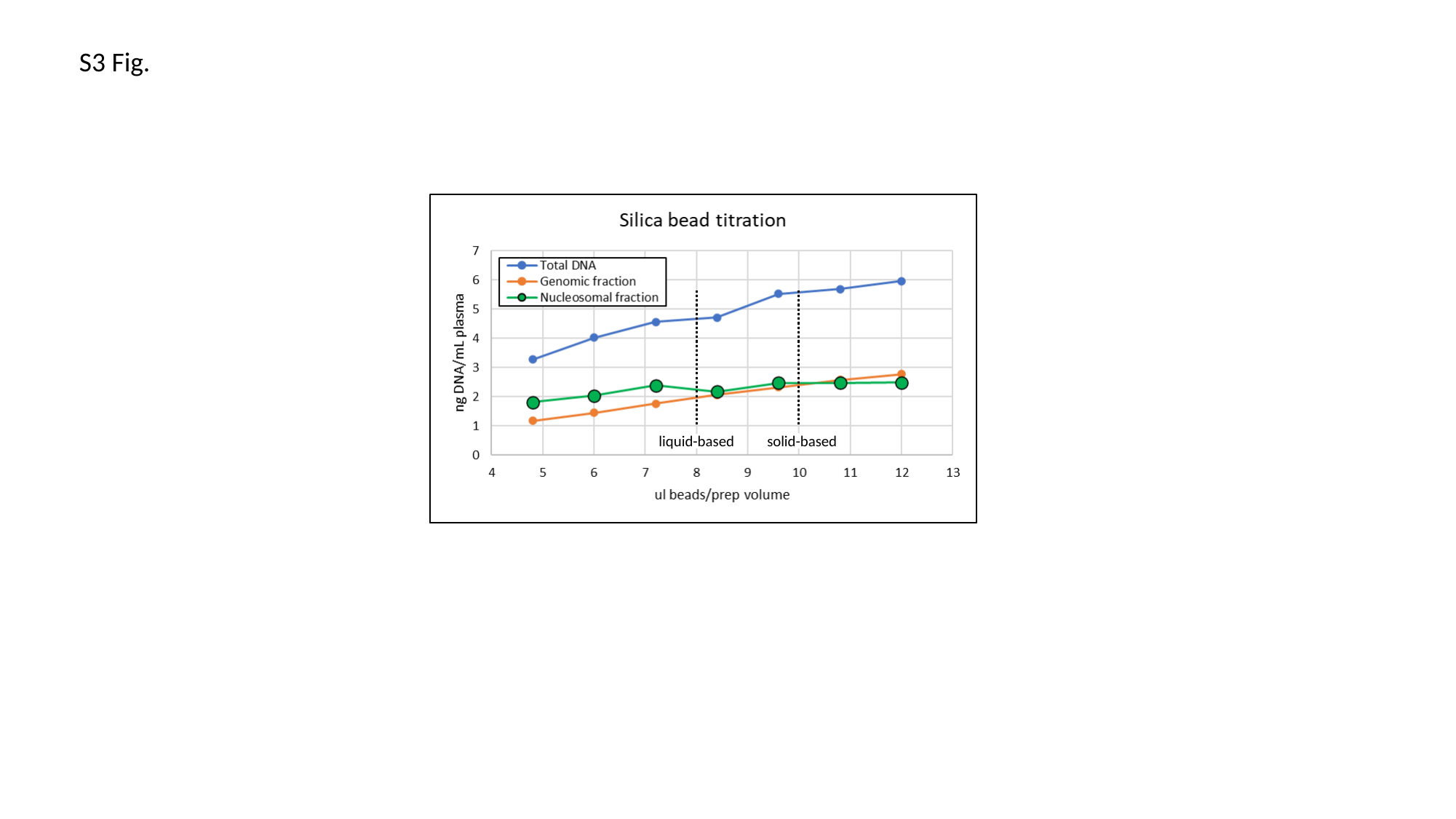

S3 Fig.
solid-based
liquid-based

Supplement: S3 Fig — Identical 10 mL aliquots of plasma were processed using the microliters of beads/prep volume shown. The yield of total DNA after the initial purification step and of high molecular weight versus nucleosomal-sized DNA is after the size selection step are shown as a function of added bead volume (2.5 mg/mL beads). The prep volume per mL of input plasma is larger for the liquid-based prep than for the solid-based prep by a factor of two-fold. In consideration of this, the quantity of 8 ul/mL prep volume was chosen for the liquid-based prep and 10 ul/mL prep volume for the solid-based prep. (PPTX) [file pone.0231854.s003.pptx]

## Slide 1
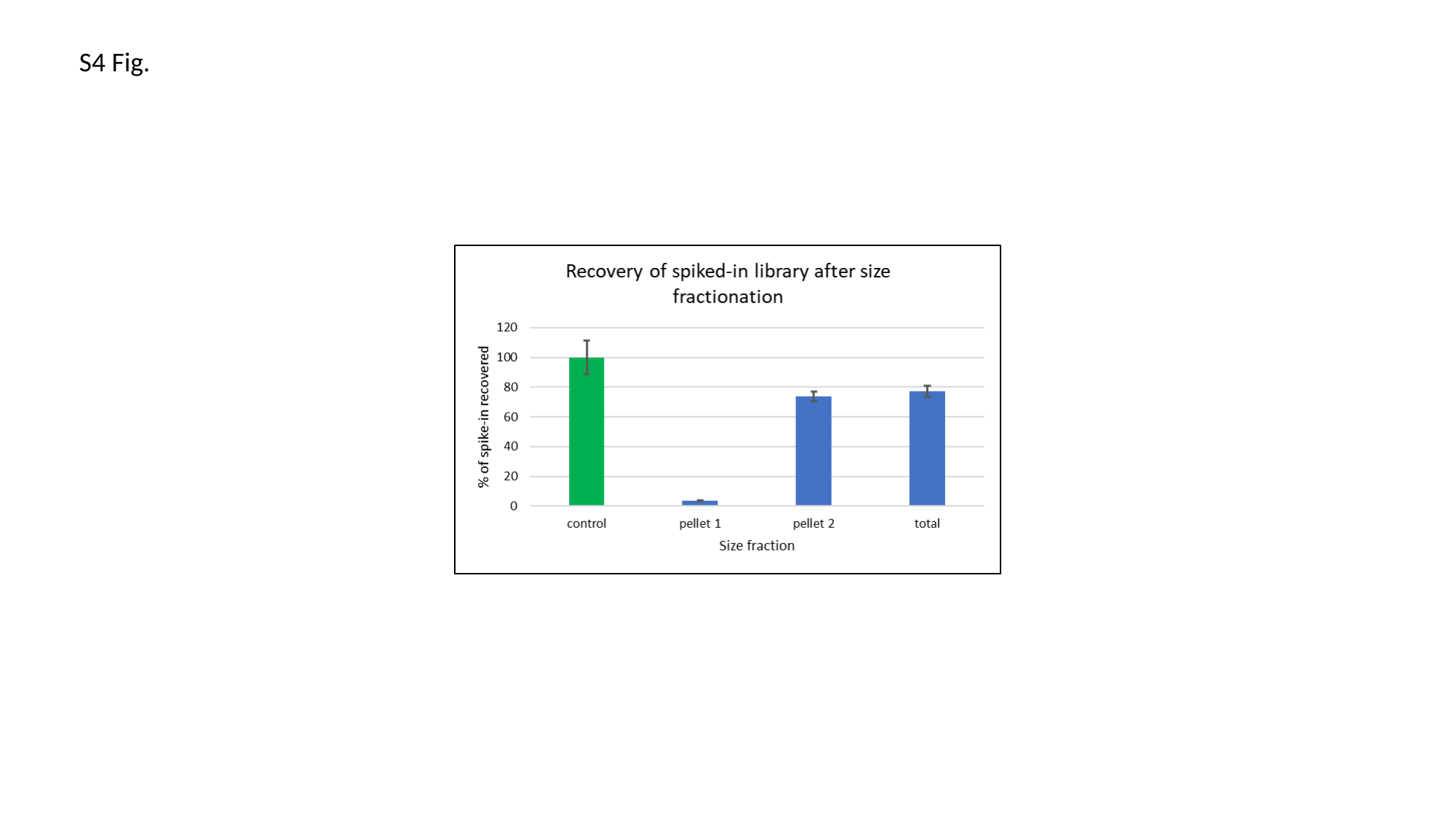

S4 Fig.

Supplement: S4 Fig — The spike-in material was completed cfDNA library with Illumina adapter sequences. Five ng per mL of total DNA was added. The amount of material eluted from the first and second bead pellets was determined by qPCR using the Illumina + Alu primer pair. “Total” recovery is the sum of the two elutions. Percentages were determined by comparison to the starting material. (PPTX) [file pone.0231854.s004.pptx]

Image used to create Fig 2

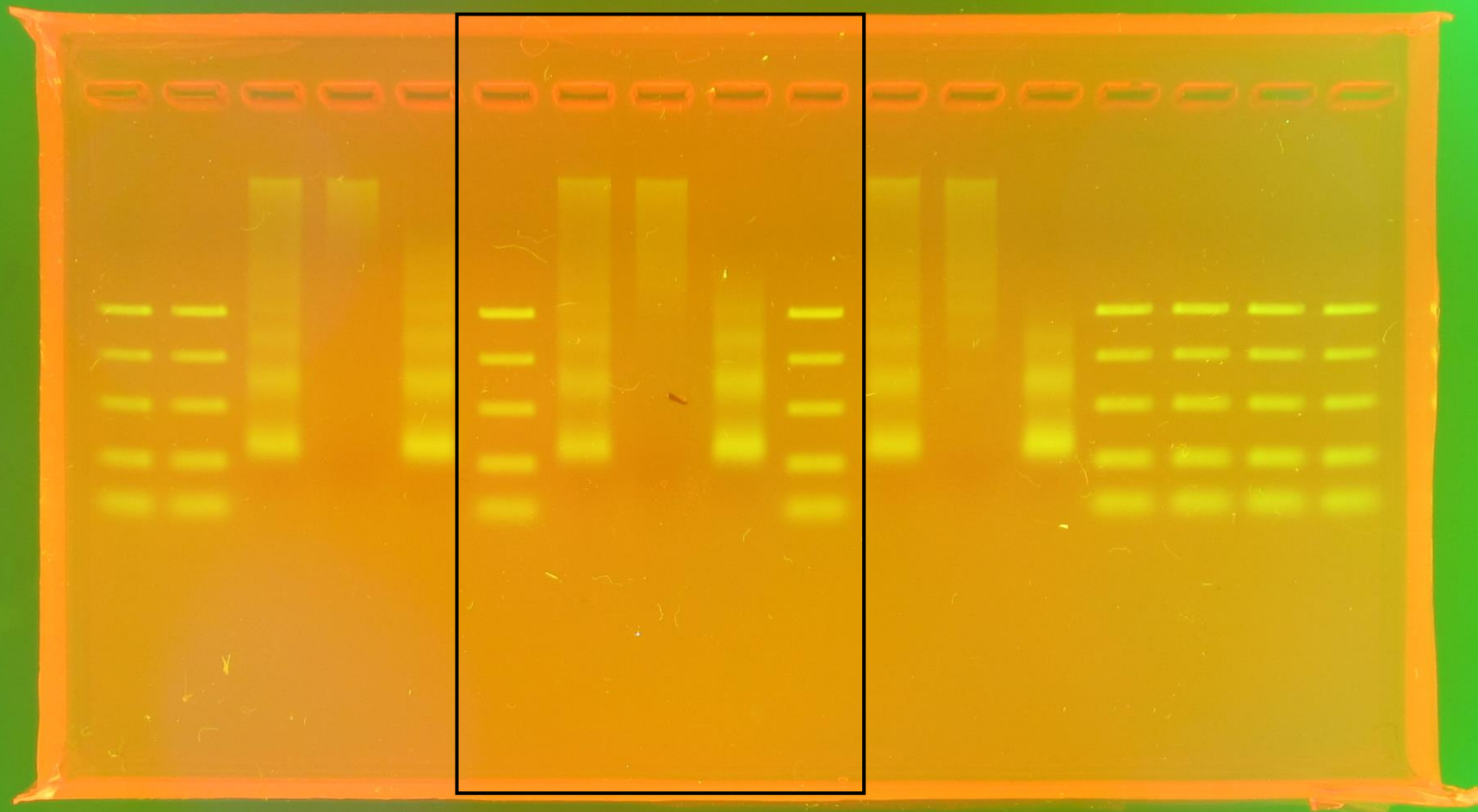

Image used to create Fig 3 and Fig 4

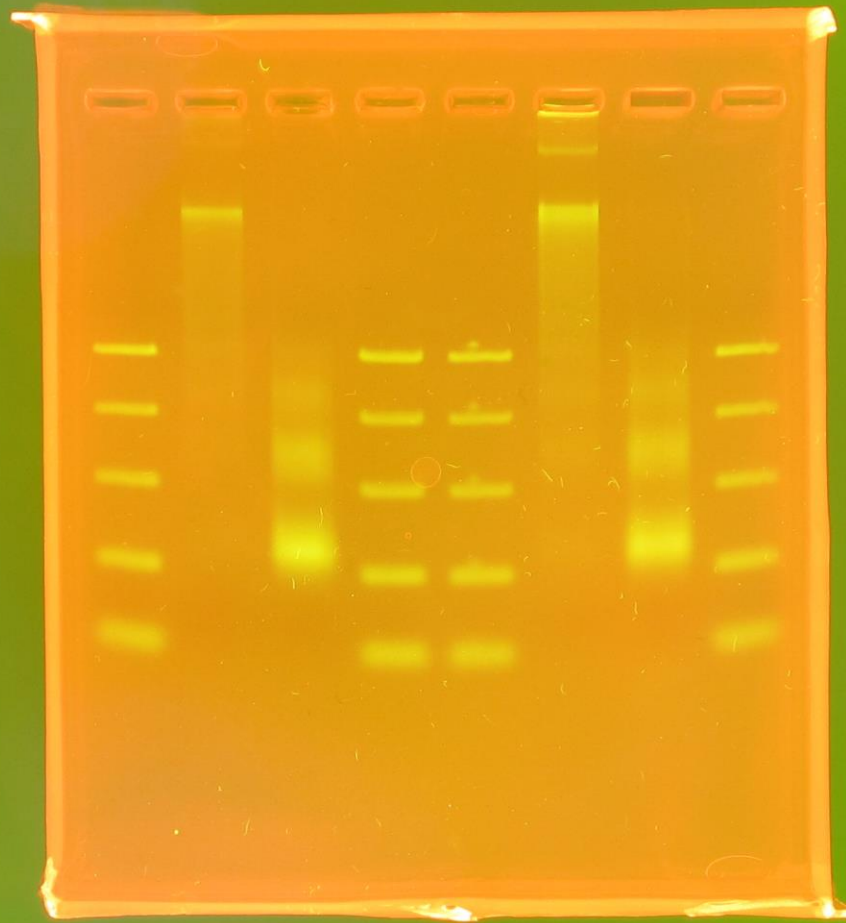

Supplement: S1 Raw images — (PDF) [file pone.0231854.s006.pdf]
