# Supplementary material for: UltraPrep is a scalable, cost-effective, bead-based method for purifying cell-free DNA
Source: PLoS One. 2020 Jun 1;15(6):e0231854. doi: 10.1371/journal.pone.0231854 (PMC7263608; doi:10.1371/journal.pone.0231854)
Supplement: Attachment — Submitted filename: 29 April 2020 Rebuttal letter.docx [file pone.0231854.s007.docx]

01 May 2020

Dear Editor Kalendar,

Thank you for consideration of our manuscript [PONE-D-20-09370] entitled “UltraPrep is a scalable, cost-effective, bead-based method for purifying cell-free DNA” authored by Christopher K. Raymond, Fenella C. Raymond and Kay Hill. We appreciate the time reviewers spent on our manuscript and invariably find that the peer-review process makes for a much stronger presentation. In addition, the methods we described will be published in protocols.io (a member of their editorial staff already contacted us), and we are awaiting a decision from PLOS ONE before moving ahead.

Please note we revised our **Funding** and **Competing Interests** statements as requested, and we added a sentence in **Competing interests** that acknowledges we derive financial benefit from the methods described in this manuscript. [*The open-source UltraPrep methods described in this manuscript are used to manufacture our commercial product, “UltraPure cfDNA”.*]

With respect to journal requirements:

- We have included the original gel photographs for gel images presented in the manuscript figures as ‘S1_raw_images’.
- **Funding:** “Funding for this study was provided jointly by Ripple Biosolutions and by PlasmaLab International. These entities provided the resources necessary to conduct this study in the form of materials, supplies and salaries. All of the authors are employees and/or owners of these entities, as detailed below. CKR and FCR are co-founders and employees of Ripple Biosolutions, and both have an ownership stake in RB. KH is an owner and director of Plasma Lab International and has an ownership stake in PLI. The funder provided support in the form of salaries for authors, but did not have any additional role in the study design, data collection and analysis, decision to publish, or preparation of the manuscript. The specific roles of these authors are articulated in the ‘author contributions’ section.”
- **Competing Interests:** All of the authors that contributed to this study are affiliated with either Ripple Biosolutions or PlasmaLab International as employees. In addition, the authors have an ownership stake in these entities. This affiliation does not alter our adherence to PLOS ONE policies on sharing data and materials. The open-source UltraPrep methods described in this manuscript are used to manufacture our commercial product, “UltraPure cfDNA”.

With respect to reviewer comments:

**Reviewer #1:**

Title of Reviewed Manuscript: UltraPrep is a scalable, cost-effective, bead-based method for purifying cell-free DNA

Detection of cell free DNA (cfDNA) in plasma currently termed “liquid biopsy” is a growing field with high importance in cancer diagnostics as well as prenatal diagnostics. The author’s approach is to use magnetic beads to separate the full amount of nucleic acid in the sample, then size select for the smaller freely circulating DNA by binding the longer DNA and using the remaining liquid with shorter fragments to bind with new beads by changing the binding conditions. This type of size selection has been demonstrated previously and is not a new innovation to the field. There is no discussion of other enrichment approaches previously reported in the literature and how this approach is different or adds value to the field.

We added three references that acknowledge the invention (as best we could determine) and the current guidance for the “two-sided SPRI” enrichment method that was incorporated into our overall work flow. Our (meager) contribution is that we provided potential readers with the exact proportions needed to separate cfDNA from genomic DNA.

The novelty of this method is the use of dry reagents added directly to plasma for large volume extraction of cfDNA. However, I would not call this approach innovative, it could be useful to those interested in extracting large volumes of plasma.

We respectfully disagree on the comments about novelty. Our most significant contribution is that we identified chemistries that enable cfDNA to bind reversibly to silica-coated magnetic beads. We attempted to capture this in Fig 1 of the paper. Several companies offer proprietary kits that claim this capability, but there is no open-source publication. This innovation enables scalability, reduces the complexity and capital requirements for execution of the protocol, and decreases the cost by about ten-fold relative to QIAamp kits (which are excellent) without sacrificing on DNA quality or the time needed for purification.

There are many concerns throughout the text with verb tense and tone, and lack of detail needed for other scientists to replicate the work. Because of this, the recommendation is to require major revisions to the manuscript.

We addressed concerns about “tense and tone” as described below. We believe that submission to protocols.io will provide the requested detail for “replication” by other scientists.

Below are specific concerns of note:

• The size separation approach has been demonstrated before in several forms and should be properly cited by the author. The dry method is interesting and perhaps useful for larger volumes of plasma. This aspect of the study is not mentioned in the abstract.

Three references for two-sided SPRI were added. We changed the wording in the abstract to indicate that “two-related protocols” are described.

• The author states that there is a “largely unmet need for large, well-characterized, single-donor lots of normal human cfDNA that can be used for diagnostic test research, assay development, and routine proficiency qualification in clinical laboratory environments.” However, it’s not clear how pre-size selected cFDNA would be helpful in these applications, unless this extraction method is used as the upfront processing for these downstream applications.

We feel the reviewer got this exactly right. We address two issues hindering cfDNA research. First, physiologically-generated cfDNA has unique characteristics that cannot be replicated by synthetic substitutes (a review article unto itself). Basic research studies and validation methods in clinical laboratories must use *bona fide* cfDNA. We believe our method provides a cost-effective solution for manufacturing this material. Second, “contamination” of circulating cfDNA with high molecular weight genomic DNA (hmwgDNA) from broken blood cells is a significant preanalytical problem (for example, it can obscure detection of rare somatic variants). The most popular approach is to use exotic blood collection tubes that are designed to mitigate cell breakage. Our alternative suggestion is to use simple size selection to enrich for the small-fragment cfDNA analyte of interest.

• Materials section:

“This seller offers an array of magnetic racks for various tube sizes that are, in our experience, the best available, and they are at a remarkably modest price point.” – Commentary is inappropriate for publication.

We changed this to “Magnetic rack bead separators were purchased from EBay (https://www.ebay.com/usr/pochekailov).”

• Methods section:

o “Digestion reagents are then added.” Only proteinase K is mentioned in previous sentence, are there more digestion reagents?

By “reagent” we meant chemicals. The text was changed from “Digestion reagents are then added” to “Digestion buffer reagents were then added.”

o Paragraph reads as an SOP, but wrong tense for manuscript: “For size selection, combine 2 volumes of total DNA with one volume of DNA purification beads [13].

Incubate for 10 min at RTo C, pull aside beads, and transfer the 3 volumes of supernatant to a vessel containing 2 additional volumes of DNA purification beads. Incubate for 10 min, pull down beads (with

bound cfDNA nucleosomal fragments), wash the bead pellet twice with an appropriate volume of 70%

ethanol/water (v/v), and resuspend in 1 ul per 1 mL of plasma (the yield in ng/ul is also the original

quantity in plasma in ng/mL).”

We corrected the protocol description from present to past tense.

• It is assumed that 100% high molecular weight DNA is removed, but only analyzed by gel electrophoresis, which has much worse sensitivity as compared to PCR. There could be some remaining and contributing to the PCR amplification signals that is just not visible by gel electrophoresis. This is a major concern.

This comment motivated us to provide additional comments about high molecular weight genomic DNA (hmwgDNA). The complete removal of hmwgDNA is not what we intended to claim. Rather, reducing the amount to quantities far less than the amount of the fragmented cfDNA is important for accurate measurement of minor allele frequencies in the setting of cancer diagnostics.

In the **Introduction** we changed:

From 1^st^ draft: This type of DNA can confound certain types of downstream genomic analysis such as quantitative digital PCR and amplicon-based DNA sequencing. There has been considerable effort invested in preanalytical collection methods that prevent cell lysis.

Revised 2^nd^ draft: When present in quantities exceeding a few percent of the total cfDNA sample that is analyzed, excess hmwgDNA can result in underestimation of minor allele frequencies for somatic DNA variants, especially when using techniques such as quantitative digital PCR or amplicon-based DNA sequencing. In the case of cancer diagnostics, these minor allele frequencies translate directly into quantitative estimates of circulating tumor DNA burden, and these in turn may be used for treatment decisions in the clinic. Hence, there has been considerable effort invested in preanalytical collection methods that prevent cell lysis.

In the **Discussion** we reiterated the point:

From 1^st^ draft: The size selection step addresses a major preanalytical concern by **removing** high molecular weight genomic DNA that can introduce artifacts into downstream analysis, especially those methods that are attempting to measure the minor allele frequency of rare tumor markers.

Revised 2^nd^ draft: The size selection step addresses a major sample collection preanalytical concern by **substantially reducing** the amount of hmwgDNA that may be present. This is significant since excess hmwgDNA can cause a significant underestimation of the minor allele frequency of rare tumor markers.

• No indication of number of replicates, error bars in any of the figures.

We added information on number of replicates and standard deviations for all of the data presented in the results section. We also added error bars to the supplemental figure data.

• All of the actual data is in supplemental figure/tables. Some of these should be in the main text, especially S1 Table. However, S1 table needs more replicates and more rigorous statistical analysis.

Dear Editor, our goal is to publish a terse paper that clearly describes our open-source method for cfDNA isolation. It is not to publish a longer manuscript detailing the development or the rigorous statistical performance characteristics of the method. The data presented in S1 Table was not intended to be a formal proof; rather, we included it because it provides some important, albeit informal, elements of analytical validation. Specifically, it shows repeatability in cfDNA yields among replicates performed on a single day, reproducibility for preparations performed on the same sample across different days, fair comparisons between our method and QIAamp (after we removed interfering carrier RNA and size fractionated the resulting DNA) and the range of cfDNA yield values across different sample types. It is our strong preference that it remain in the paper as supplemental information.

• There is PEG mentioned in the text, but not in any of the method details or Tables. Unclear when and how this method was used in the study.

We changed the wording in the Results section to make it clear that polyethylene glycol (PEG) is a constituent of the DNA purification bead solutions that are used for DNA fragment size selection. In the original draft of the manuscript we provided a brief description of double-sided SPRI for the benefit of readers that may be unfamiliar with the concepts. We feel this description should remain in the revised manuscript.

• The reviewer was unable to find these measurements of “amplifiability” and “clonability”reported later in the text.

“We used qPCR measurement of human Alu sequences to establish that the specific “amplifiability” per picogram of purified cfDNA was consistent between purified lots of material and similar to cfDNA purified using the industry standard (QIAamp). Fourth, quantitative NGS methods for cfDNA analysis rely on the attachment of adapter sequences as a prerequisite for creating genomic cfDNA libraries. We monitored the “clonability” of purified cfDNA by measuring the attachment efficiency of adapters containing standard Illumina sequences to cfDNA.”

We believe that the reviewer’s objection to tone (“There are many concerns throughout the text with verb tense and tone,”) center around the *ad hoc* phrases “amplifiability” and “clonability”. We apologize for inventing phrases rather than using more scientifically acceptable descriptions. We eliminated “amplifiability” and replaced it with “assay to detect PCR inhibition”. We eliminated “clonability” and replaced it with “The percentage of cfDNA ends that become ligated an adapter is often referred to as the “conversion rate””. Note we did not invent the phrase “conversion rate”, rather it is commonly used in the NGS literature.

**Reviewer #2:**

The paper describes a new open-source, two-step method for purification of cell-free DNA. There is a high demand in non-expensive efficient alternative approaches for cell-free DNA isolation for research purposes, and a publication may attract particular interest in this regard.

Given that reproducibility of the method suggested is the main value of the publication, the 'Materials' section could be enforced by more specific information about the items used (like Cat# or type of silica-coated superparamagnetic beads). For non-standardized items from unknown sellers (like pochekailov), quality alternatives from recognized sellers could be suggested.

We included the specific information about proteinase K and silica beads in the Materials section. For all other reagents, the vendor we cite only have one product choice.

*Please allow us a personal note on magnetic racks. Several reviewers objected to the initial “infomercial” language and we altered the text accordingly. We have no financial stake in Sergii Pochekailov’s business. The first author did work with Sergii to design the optimal racks for our application, and he now sells the same design on EBay. These racks are the best tools we have found for the process we describe, and they are incredibly inexpensive. To reinforce reproducibility for your readers, we felt compelled to say, “Look here first.”*

We added the sentence, “We have not tested the performance of alternative 50 mL magnetic racks, but they appear to be widely available.”

Plasma separation and storage conditions may play a key role in quality cfDNA extraction, so authors are suggested to add this information about conditions used in their experiments.

We added a significant amount of descriptive detail about plasma collection and storage in the Materials section.

“Plasma samples were collected at PlasmaLab International (Everett, WA) using automated plasmapheresis collection into sodium citrate containing collection vessels. The samples used for protocol development and described in S1 Table were stored at -20^o^ C for as long as 10 years. The K_2_EDTA samples were derived from whole blood collected in standard vacutainer tubes that was spun immediately at 1500 g for 10 min. The top plasma fraction was transferred to a separate container and frozen at -20^o^ C prior to the cfDNA preparation.”

Although some comparison with the leader on a market has been performed, the manuscript will certainly benefit if authors are following one of the formal standardized frameworks for validation of the molecular tests (like https://www.ncbi.nlm.nih.gov/pmc/articles/PMC3002854/, but more could be available).

The title of the publication the reviewer recommended was “A standardized framework for the validation and verification of clinical molecular genetic tests.” We are describing what is essentially a *research-use-only* method for DNA purification. These are wildly different topics. We were not able to translate this comment into a meaningful revision of the manuscript.

I am not aware of Research Ethics procedures in commercial organizations and cannot comment on this topic.

At the recommendation of PLOS ONE editors, we used this language in a previous publication (PLoS One. 2017 Apr 27;12(4):e0176241). The key point is that plasma donors signed a consent form prior to plasma collection that has been vetted by the FDA.

**Reviewer #3:**

*Many thanks to this reviewer for the detailed review!*

Raymond and co-workers authors describe a method for purification of cfDNA that includes selective enrichment of smaller fragments thought to derive from cleavage of human DNA between nucleosomes of apoptotic cells.

Two protocols have been described, for smaller and larger input plasma volumes. The authors compared the performance of the UltraPrep metrics for cfDNA purified from the same plasma using the QIAamp procedure. The cfDNA obtained by the two methods generated similar quality metrics but with 10 times lower cost for UltraPrep.

Here below are my comments:

Page 2

Another potential domain of application of cfDNA is detection of bacterial pathogens (bacteraemia, sepsis). This may be mentioned in the introduction.

We added this sentence, “This analyte has also shown utility for the rapid detection of infectious microbes [3].” The reference we cite is the first and most cited article that shows up on PubMed.

“… preanalytical collection conditions often result in plasma that is a mixture of high molecular weight genomic DNA and cfDNA”. This sentence is slightly unclear.

Perfect catch! We meant to say “plasma that contains” and altered the text accordingly.

“This seller offers an array of magnetic racks for various tube sizes that are, in our experience, the best available, and they are at a remarkably modest price point. “ I would remove this sentence (looks like an advertisement).

This has been addressed.

Page 5, Table 1

Please check if 400 nM (nanomolar) Silica beads is correct (also in Table 2). Or you meant 400-nm diameter?

We changed nM to nm.

Is proteinase K at 1/50 plasma volume? Or 1/100?

1/100 (corrected)

Silica beads are at 1/100 or 1/125 volume?

1/125 (corrected)

Remove “.” in “TE Buffer”/“Notes” cell; add the degree symbol where required; if you capitalise the first letter of “isopropanol” than apply the same for “glycerol”.

Corrected in Table 1 and Table 2

Page 6, Table 2. The text should be revised. In “Plasma”/”Notes” delete “cf DNA purification”.

Deleted.

“Digestion step”/“Notes” may be clarified. For instance, If I correctly understood: “Mix the 4 components and heat in a hot water bath at XXX °C to dissolve GITC and Tween 20. Cool to 56 °C, mix with plasma/proteinase K and incubate at 56 °C for 1 hour. (“Monitor temperature and do not exceed 56 C.” may be deleted). Clarify if the components are mixed together prior to adding to plasma/proteinase K or they are added sequentially. If mixed, merge the 4 cells “Cumulative volume” for the 4 digestion reagents (140 mL).

We corrected the cell merge issue in two places and clarified the accompanying notes.

In “TE Buffer”/”Notes” add “eluate” after “DNA”.

Corrected

grams ---> g

Corrected

Do 50-mL-tubes accommodate 50 mL + 5 mL (Wash 1) + 5 mL (Wash 2) + 1 mL (ethanol) volume?

Changed notes in Table 2 to clarify

Page 9, Caption Fig 4.

“Comparison of Alu units per pg “. “Please indicate what is measured in pg (e.g. ”pg of XXX”).

Changed to “Comparison of Alu yields per ng of cfDNA”

Page 10, Discussion

In addition to cfDNA purification, plasmapheresis and downstream sequencing or ddPCR generate costs. What percentage of the overall cost is due to cfDNA purification with Qiagen and UltraPrep approaches?

We think this is a great question but it is beyond the scope of our manuscript (could be the topic of endless debate among Reviewers). As an aside, the goal that inspired this project was to reduce the cost of extracting cfDNA from one liter of plasma (which can be obtained for <$1000/liter) from >$6000 using QIAamp to something far less.

What does UltraPure correspond to? Corrected to “Ultraprep”

Figure 2 - The figure would be easier to read if you indicate the content of tubes A, B and G directly in the figure.

Typos

“comnparison” on page 8. Corrected

RT° C ---> room temperature (RT). Corrected

Use mL (or ml) but not both. Corrected

mLs ---> mL. Corrected

In tables and figures, add space before “M“ (molar) or “mM”. Corrected

**Reviewer #4:**

The authors attempt to describe a novel method for purifying small sized nucleic acids from blood and plasma samples. While this is an interesting proposition, the manuscript is not written in a manner consistent with a scientific level publication. Namely, key data from experiments simply is not presented. As such I am unable to fully assess its scientific merits. I want to underscore that any and all data need to presented - key data should be in the manuscript itself and any additional data in the supplemental material. I have highlighted this, along with other points below. One note, please check the supplemental material files - my Powerpoint told me the files are corrupted.

These comments sound similar to those expressed by Reviewer #1. In response we corrected errors in tense, used more formal language in descriptions, added data with error analysis to the results, and provided error bars in supplemental figures.

Summary: RESULTS

1. “First, proteinase K is often the most expensive reagent used in DNA extraction procedures”

a. S1 Fig shows that the lowest concentration was already giving the best results. Why not try lower doses?

b. Others have reported that Proteinase K may not even be needed: http://pubmed.gov/28923054. The main difference here may be the use of a final conc of 2% Triton. If cost is really a concern, it may be worth doing a quick experiment to see if Prot K can be left out. This may have the tangential benefit of preserving the small nuc acids which may degrade during Prot K treatment and reducing the length of the protocol.

It is unfortunate that the files examined by this reviewer were corrupted, because the actual figure clearly indicates that we performed the no enzyme controls. These no enzyme controls were difficult to work with and we cannot recommend a no enzyme protocol. We chose the amount of enzyme based on cost; lower amounts did not represent substantial additional savings and the no enzyme controls show that some enzyme is beneficial. We added the following to the S1 figure legend:

“This amount of enzyme corresponds to four cents per mL of processed plasma, which is less than 10% of the overall cost. While there were significant yields of DNA in the no-added-enzyme control samples, these samples were “sticky” and extremely difficult to process.”

2. “Fourth, the method is scalable, meaning the yield of extracted DNA per milliliter of input is consistent across a broad range of sample input volumes.”

a. Was this shown experimentally?

In the S1 Table, we clearly indicated whether samples were processed by the liquid prep method, the solid prep method or the QIAamp method. We added the following clarifying sentence to the end of the second paragraph of the results section:

“Both methods produce comparable yields of cfDNA (S1 Table).”

3. “Coincidentally, in the plasmapheresis samples we have worked with, about 1/3 of the total DNA is high molecular weight and 2/3 is nucleosomal cfDNA (for example, see Fig 2).”

a. Show the data that backs up this statement

This point is presented visually in Fig 2. We would be happy to state “data not shown”, to delete the sentence altogether, or to leave it as the helpful observation it was intended to be.

4. Fig 3:

a. Where’s the data?

The data is in Fig 4.

5. “First, the QIAamp kit is a total DNA isolation method that collects both high molecular weight genomic DNA and cfDNA fragments”

a. This data should be shown.

It is not our job to show the DNA size collection specifications for Qiagen’s QIAamp mini spin columns. This information is readily available.

6. “Second, we found that the carrier RNA routinely added during QIAamp purification is a significant interfering substance”

a. The Qiagen handbook states that “carrier RNA present in the extracted nucleic acids is likely to dominate UV absorbance readings”, so this is already known. But this shouldn’t be a factor because as stated in the methods section, eluted samples are treated with RNAse and the dye used for Qubit is “dsDNA-specific”. Please explain.

We feel we were quite clear on this point. First, we found that carrier RNA overinflates DNA readings when using a Qubit fluorometer and dsDNA-specific dyes. Second, we confirmed this by pretreating the Qiagen samples with RNAase (which is not a strategy covered in the Qiagen manual). The key result was that the DNA yield from either our method or QIAamp were similar once this was done. RNAase treatment is not a standard part of our protocol (since we don’t need to add RNA carrier). Our goal was to be as fair as possible in our evaluation of the QIAamp, which is an excellent product.

7. Performance section:

a. Please present the data

We added more data, error analysis and number of replicates. The key data is clearly presented in Fig 4.

METHODS

1. Two additional Supplemental files should be provided:

a. 1) A Word document that outlines each protocol in detail (ie step by step)

b. 2) A Excel table that lists the reagents and equipment used for the protocols (along with corresponding catalogue #)

In response to comment a., we intend to work with protocol.io if the paper is accepted. In response to comment b. we modified the Materials section with specific detail about the two most critical components, proteinase K and silica-coated beads.

2. A suitable replacement from a commercial vendor needs to be given for the item from Ebay as labs in some countries may not be able to procure from Ebay

This point has been addressed.

3. Please explain in more detail regarding your spike-in libraries. I could not fully understand how you carried out these experiments and how you made the comparisons for percent recovery.

We added detail to the S2 Fig legend describing the spike-in DNA and the fact that it can be uniquely detected using one of the primer pairs described in the Methods section.

OTHER:

1. “comnparison” – typo Corrected.

2. Show DNA ladder sizes on your figures Done

3. Show proper statistics, including standard deviations (I’m assuming experiments were done in replicates and at least 2 independent times) Addressed

4. What are you staining you gels with? Is it a ds-DNA specific stain or does it also stain RNA? As mentioned in the Methods section, DNA gels were stained with Gel Green (Biotium). We added mention of this in two additional places (once in Materials and once more in Methods). The manufacturer states that Gel Green can stain RNA with poor sensitivity.
