# Supplementary material for: UltraPrep is a scalable, cost-effective, bead-based method for purifying cell-free DNA
Source: PLoS One. 2020 Jun 1;15(6):e0231854. doi: 10.1371/journal.pone.0231854 (PMC7263608; doi:10.1371/journal.pone.0231854)
Supplement: Attachment — Submitted filename: 14 May 2020 Response to reviewer #4.docx [file pone.0231854.s008.docx]

14 May 2020

**Subject:**

PONE-D-20-09370R1

UltraPrep is a scalable, cost-effective, bead-based method for purifying cell-free DNA

Christopher K. Raymond, Fenella C. Raymond and Kay Hill

[craymond@ripplebiosol.com](mailto:craymond@ripplebiosol.com) or [chrisk.kraymond@gmail.com](mailto:chrisk.kraymond@gmail.com)

cell: 206-715-4527

Dear Editor Kalendar,

Thank you for the continuing opportunity to work with PLOS ONE. We are eager to address the concerns of Reviewer #4 and look forward to publication of our manuscript in PLOS ONE.

From a broad perspective, our goal is to present an open-source method for purification of circulating cell-free DNA (cfDNA) from human plasma (and more generally, possibly other biofluids). This diagnostic analyte is central for noninvasive prenatal testing and for genetic analysis of advanced solid cancers. It may become the cornerstone for early cancer detection, pathogen identification, chronic disease monitoring, and tissue transplant rejection monitoring. While there is a plethora of (expensive) kits for cfDNA purification, to our knowledge there are no (recent) published methods. Hence, we believe this article will be of significant interest to your readership.

Our primary focus was to provide a clear description of the protocols. We followed the model set forth in another PLoS article (Oberacker P, Stepper P, Bond DM, Höhn S, Focken J, Meyer V, et al. Bio-On-Magnetic-Beads (BOMB): Open platform for high-throughput nucleic acid extraction and manipulation. PLoS Biol. 2019 Jan 10;17(1)). As with that publication, we provide graphical protocols (a picture is worth a thousand words), a set of Tables that provide step-by-step information, and a brief written description. The idea was to provide full transparency by conveying information in multiple formats such that we could implement the method in our own laboratory.

Our secondary focus was to provide a framework for evaluating purified cfDNA and to compare our method to Qiagen’s QIAamp Circulating Nucleic Acids purification kit that is the “gold-standard” for cfDNA purification. We went to great lengths to cast QIAamp in the most favorable light possible, given that it is an excellent “cornerstone” product.

Finally, the supplemental data was included to 1) provide a real-world glimpse of reproducibility and repeatability, 2) support claims on the percent yields from various steps in the protocol and 3) support our recommended amounts of the most expensive components used (silica beads and proteinase K).

Our sense is that the manuscript provides a complete description of the method that will allow other scientists to evaluate the procedure and determine its merit in their own hands. We believe we provide sufficient documentation to support our claims without being overly detailed.

With respect to Reviewer #4, these are the second set of comments we received:

Comments to the Author

Reviewer #4: (No Response)

2. Is the manuscript technically sound, and do the data support the conclusions?

Reviewer #4: Partly

3. Has the statistical analysis been performed appropriately and rigorously?

Reviewer #4: No

4. Have the authors made all data underlying the findings in their manuscript fully available?

Reviewer #4: No

5. Is the manuscript presented in an intelligible fashion and written in standard English?

Reviewer #4: Yes

6. Review Comments to the Author

Reviewer #4:

Authors have not sufficiently addressed my concerns. I cannot in good faith recommend this article for publication. (The authors should know that even though the files were deemed corrupted by my Powerpoint, I was able to open them as google docs and was able to assess their quality.)

Editor, in the absence of specific comments, we found it difficult to make additional revisions. In particular:

- 2. We believe we provided a complete description of the two related methods (technically sound) and sufficient data to validate our claims (data support the conclusions).
- 3. The first set of comments from Reviewer 4 asked for averages and standard deviations. We provided these.
- 4. We’re not sure what “data underlying the findings” we left out.

These are our initial comments in response to Reviewer #4 that were sent with the first revision. As we reread them, we feel we addressed this reviewer’s specific concerns in our initial revision effort.

**Reviewer #4:**

The authors attempt to describe a novel method for purifying small sized nucleic acids from blood and plasma samples. While this is an interesting proposition, the manuscript is not written in a manner consistent with a scientific level publication. Namely, key data from experiments simply is not presented. As such I am unable to fully assess its scientific merits. I want to underscore that any and all data need to presented - key data should be in the manuscript itself and any additional data in the supplemental material. I have highlighted this, along with other points below. One note, please check the supplemental material files - my Powerpoint told me the files are corrupted.

These comments sound similar to those expressed by Reviewer #1. In response we corrected errors in tense, used more formal language in descriptions, added data with error analysis to the results, and provided error bars in supplemental figures.

Summary: RESULTS

1. “First, proteinase K is often the most expensive reagent used in DNA extraction procedures”

a. S1 Fig shows that the lowest concentration was already giving the best results. Why not try lower doses?

b. Others have reported that Proteinase K may not even be needed: http://pubmed.gov/28923054. The main difference here may be the use of a final conc of 2% Triton. If cost is really a concern, it may be worth doing a quick experiment to see if Prot K can be left out. This may have the tangential benefit of preserving the small nuc acids which may degrade during Prot K treatment and reducing the length of the protocol.

It is unfortunate that the files examined by this reviewer were corrupted, because the actual figure clearly indicates that we performed the no enzyme controls. These no enzyme controls were difficult to work with and we cannot recommend a no enzyme protocol. We chose the amount of enzyme based on cost; lower amounts did not represent substantial additional savings and the no enzyme controls show that some enzyme is beneficial. We added the following to the S1 figure legend:

“This amount of enzyme corresponds to four cents per mL of processed plasma, which is less than 10% of the overall cost. While there were significant yields of DNA in the no-added-enzyme control samples, these samples were “sticky” and extremely difficult to process.”

2. “Fourth, the method is scalable, meaning the yield of extracted DNA per milliliter of input is consistent across a broad range of sample input volumes.”

a. Was this shown experimentally?

In the S1 Table, we clearly indicated whether samples were processed by the liquid prep method, the solid prep method or the QIAamp method. We added the following clarifying sentence to the end of the second paragraph of the results section:

“Both methods produce comparable yields of cfDNA (S1 Table).”

3. “Coincidentally, in the plasmapheresis samples we have worked with, about 1/3 of the total DNA is high molecular weight and 2/3 is nucleosomal cfDNA (for example, see Fig 2).”

a. Show the data that backs up this statement

This point is presented visually in Fig 2. We would be happy to state “data not shown”, to delete the sentence altogether, or to leave it as the helpful observation it was intended to be.

4. Fig 3:

a. Where’s the data?

The data is in Fig 4.

5. “First, the QIAamp kit is a total DNA isolation method that collects both high molecular weight genomic DNA and cfDNA fragments”

a. This data should be shown.

It is not our job to show the DNA size collection specifications for Qiagen’s QIAamp mini spin columns. This information is readily available.

6. “Second, we found that the carrier RNA routinely added during QIAamp purification is a significant interfering substance”

a. The Qiagen handbook states that “carrier RNA present in the extracted nucleic acids is likely to dominate UV absorbance readings”, so this is already known. But this shouldn’t be a factor because as stated in the methods section, eluted samples are treated with RNAse and the dye used for Qubit is “dsDNA-specific”. Please explain.

We feel we were quite clear on this point. First, we found that carrier RNA overinflates DNA readings when using a Qubit fluorometer and dsDNA-specific dyes. Second, we confirmed this by pretreating the Qiagen samples with RNAase (which is not a strategy covered in the Qiagen manual). The key result was that the DNA yield from either our method or QIAamp were similar once this was done. RNAase treatment is not a standard part of our protocol (since we don’t need to add RNA carrier). Our goal was to be as fair as possible in our evaluation of the QIAamp, which is an excellent product.

7. Performance section:

a. Please present the data

We added more data, error analysis and number of replicates. The key data is clearly presented in Fig 4.

METHODS

1. Two additional Supplemental files should be provided:

a. 1) A Word document that outlines each protocol in detail (ie step by step)

b. 2) A Excel table that lists the reagents and equipment used for the protocols (along with corresponding catalogue #)

In response to comment a., we intend to work with protocol.io if the paper is accepted. In response to comment b. we modified the Materials section with specific detail about the two most critical components, proteinase K and silica-coated beads.

2. A suitable replacement from a commercial vendor needs to be given for the item from Ebay as labs in some countries may not be able to procure from Ebay

This point has been addressed.

3. Please explain in more detail regarding your spike-in libraries. I could not fully understand how you carried out these experiments and how you made the comparisons for percent recovery.

We added detail to the S2 Fig legend describing the spike-in DNA and the fact that it can be uniquely detected using one of the primer pairs described in the Methods section.

OTHER:

1. “comnparison” – typo Corrected.

2. Show DNA ladder sizes on your figures Done

3. Show proper statistics, including standard deviations (I’m assuming experiments were done in replicates and at least 2 independent times) Addressed

4. What are you staining you gels with? Is it a ds-DNA specific stain or does it also stain RNA? As mentioned in the Methods section, DNA gels were stained with Gel Green (Biotium). We added mention of this in two additional places (once in Materials and once more in Methods). The manufacturer states that Gel Green can stain RNA with poor sensitivity.

Thank you for your consideration and we look forward to a response.

Sincerely,

Chris Raymond
